# Improving gross anatomy learning using reciprocal peer teaching

**DOI:** 10.1186/s12909-016-0617-1

**Published:** 2016-03-22

**Authors:** Mange Manyama, Renae Stafford, Erick Mazyala, Anthony Lukanima, Ndulu Magele, Benson R. Kidenya, Emmanuel Kimwaga, Sifael Msuya, Julius Kauki

**Affiliations:** Department of Anatomy and Cell Biology, Catholic University of Health and Allied Sciences, Mwanza, Tanzania; Department of Surgery, Catholic University of Health and Allied Sciences, Mwanza, Tanzania; Department of Biochemistry and Molecular Biology, Catholic University of Health and Allied Sciences, Mwanza, Tanzania; Kilimanjaro Christian Medical University College, Moshi, Tanzania

## Abstract

**Background:**

The use of cadavers in human anatomy teaching requires adequate number of anatomy instructors who can provide close supervision of the students. Most medical schools are facing challenges of lack of trained individuals to teach anatomy. Innovative techniques are therefore needed to impart adequate and relevant anatomical knowledge and skills. This study was conducted in order to evaluate the traditional teaching method and reciprocal peer teaching (RPT) method during anatomy dissection.

**Methods:**

Debriefing surveys were administered to the 227 first year medical students regarding merits, demerits and impact of both RPT and Traditional teaching experiences on student’s preparedness prior to dissection, professionalism and communication skills. Out of this, 159 (70 %) completed the survey on traditional method while 148 (65.2 %) completed survey on RPT method. An observation tool for anatomy faculty was used to assess collaboration, professionalism and teaching skills among students. Student’s scores on examinations done before introduction of RPT were compared with examinations scores after introduction of RPT.

**Results:**

Our results show that the mean performance of students on objective examinations was significantly higher after introduction of RPT compared to the performance before introduction of RPT [63.7 ± 11.4 versus 58.6 ± 10, mean difference 5.1; 95 % CI = 4.0–6.3; *p*-value < 0.0001]. Students with low performance prior to RPT benefited more in terms of examination performance compared to those who had higher performance [Mean difference 7.6; *p*-value < 0.0001]. Regarding student’s opinions on traditional method versus RPT, 83 % of students either agreed or strongly agreed that they were more likely to read the dissection manual before the RPT dissection session compared to 35 % for the traditional method. Over 85 % of respondents reported that RPT improved their confidence and ability to present information to peers and faculty compared to 38 % for the tradition method. The majority of faculty reported that the learning environment of the dissection groups was very active learning during RPT sessions and that professionalism was observed by most students during discussions.

**Conclusions:**

Introduction of RPT in our anatomy dissection laboratory was generally beneficial to both students and faculty. Both objective (student performance) and subjective data indicate that RPT improved student’s performance and had a positive learning experience impact. Our future plan is to continue RPT practice and continually evaluate the RPT protocol.

## Background

The increase in the number of students’ intake in medical schools across the world has added upon the challenges in teaching Human Anatomy as a medical and clinical science [1]. In most medical schools in Africa, including at the Catholic University of Health and Allied Sciences (CUHAS), anatomical skills and knowledge are still being gained primarily through didactic lectures and complete dissection of the human body [2–4]. The use of cadavers in human Anatomy teaching requires an adequate number of Anatomy instructors who can provide close supervision of the students [5]. However, most schools across the world are facing shortage of qualified anatomists [6–11]. The department of Anatomy at CUHAS is estimated to have a shortage of teaching staff of about 43 % [7]. The shortage of qualified anatomists has forced training institutions to design various strategies aimed at educating the next generation of health professionals including, team-teaching gross anatomy, allow postdoctoral fellows to participate in teaching etc. [6]. Reduction in time allocated for teaching anatomy has widely been reported among medical schools [1, 5, 12]. In many parts of the world, the practice of teaching anatomy through didactic lectures and complete dissection of the body has been minimized in order to streamline anatomy teaching with integration of clinical sciences [5, 13, 14]. This has been done through the addition of special study modules, integration of anatomy in problem-based learning, the use of prosected plastinated specimens, computer-generated images, plastic models and other teaching tools [13, 14]. The combination of increase in students’ intake, reduction in allocated time for Anatomy and shortage of Anatomy teachers are likely to lead to tomorrow’s surgeons and physicians lacking the detailed knowledge of Anatomy and potentially a questionable professional capability [15].

Traditionally, Anatomy in most medical schools has been taught at the beginning of medical education to provide a basis for clinical training and practice [1]. Despite being replaced by two- or three-dimensional virtual representations and computer-assisted learning programs, cadaver dissection has remained core to Anatomy teaching in most parts of the world [2–4, 16, 17]. Dissection of the human cadaver instructs not only in structure and function, but also introduces medical students to the basic language of medicine, learning in peer groups and how to function as part of a team [15, 18]. It is in the dissection laboratory where students form their ideas and mental images of the structure of the human body at different levels over time. In recent years, some medical schools have replaced dissection with two- or three-dimensional virtual representations and computer-assisted learning programs as to resolve challenges associated with teaching Anatomy such as a shortage of anatomists [5, 18–20]. It’s argued however, that Anatomy course objectives in medical education cannot be fulfilled by the use of two- or three-dimensional virtual representations and computer-assisted learning programs alone without the use of dissection [21]. Indeed, many medical educationalists hold the view that lessons learnt from the actual feel of human tissue are unsurpassed [22, 23]. The use of modern technology like computers, however advanced they may be, will not match the use of real cadavers in dissection laboratories because they can never equate with the complex and miraculous reality of a human body [24]. Integration of newer teaching modalities and modern technology will encourage interest and retention of anatomical knowledge and its clinical relevance [1].

The Anatomy course for medical students at CUHAS is offered through a traditional teacher-oriented teaching method where students attend lecture classes that are followed by the dissection of cadavers. This is usually done during the first semester of medical school. One advantage of the teacher-centered learning is that it allows the instructor to determine the aims, content, organization, pace and direction of a presentation [25]. However, teacher-centered learning encourages one-way communication and hence making it difficult for lecturer to become aware of student academic weaknesses [25, 26]. The other drawback of teacher-centered is that it places students in a passive rather than an active role, which hinders learning [26]. Student-centered learning methods enables students to direct their own learning, promotes student collaboration and communication through group work and identifies a “hidden curriculum” [27, 28]. However, student-centered approach can sometimes be seen as learning with not as much structure or discipline as a traditional method, causing student’s to feel overwhelmed and sometimes leading to impaired completion of objectives [27, 28]. In order to increase their accountability to communities, there is a huge drive for medical schools to promote student-centred and problem-oriented approaches which produce doctors better equipped with the adult learning skills necessary for them to adapt to, and meet, the changing needs of the community they serve [27].

Dissection is the dominant learning mode in terms of time allocation, with approximately 60 % of the total teaching time allocated to human cadaver dissection. It is therefore critical that the time allocated for dissection is utilized effectively in order to give students a hands-on view of the body while also appreciating the three dimensions of anatomical structures. The dissection activity is not only an opportunity for self learning but also a forum at which students discuss among themselves and assist in each other’s education [29, 30]. With the current shortage of Anatomy faculty at CUHAS, it is not possible for medical students to understand the subject conceptually in the scheduled course duration. Thus, additional innovative ways are needed in this traditional teacher-oriented training system in order to improve student’s learning.

Reciprocal Peer Teaching (RPT) wherein students alternate roles as teacher and learner is a collaborative approach that embeds assessment in a formalized learning process to facilitate student involvement with course content and improve achievement [31]. The term reciprocal peer teaching (RPT) was first proposed in 1978 by Allen and Boracks to illustrate circumstances where students alternate roles as teacher and student [32]. RPT is based on the philosophy that ‘those who teach learn’. Under this educational paradigm, students vary their roles, being alternately charged with teaching their peers, and then being instructed in kind. The dual responsibilities experienced during RPT, enables students to learn both from the preparation and tutor’s engagements and from the instructions that the tutees receive [33, 34]. By involving learners in the responsibility for their own learning and that of others, RPT transforms learning from private to a social activity [34]. RPT approach has been applied to gross Anatomy education with results showing that it leads to improved learning [35–39]. Other benefits of RPT over the other similar teaching protocols include promotion of student’s active learning through direct interactions, greater understanding resulting from sharing similar discourse between peers and students and reinforcement of peer teacher’s learning through instructing others [37, 38]. In addition, RPT is financially efficient alternative to hiring more staff members [31–33].

**Fig. 1:**
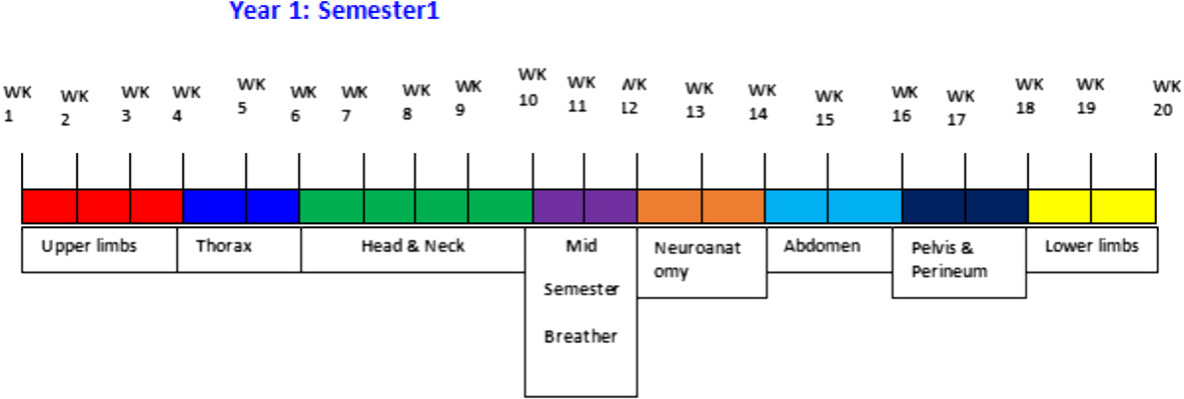
Anatomy Teaching Schedule at CUHAS. A schematic diagram showing a teaching schedule for gross anatomy for first year medical students

Traditionally, cadaver dissection at CUHAS and most of other medical schools in Tanzania has been conducted by students with assistance from anatomy faculty. Students are divided into groups of 15–20 depending on the size of the class and the number of cadavers available. At the time this study was conducted, the total number of students and anatomy faculty were 227 and 6 respectively, making a faculty:student ratio of 1:38. A two-hour lecture on the region to be dissected is usually given on the morning and a three-hour dissection session is performed in the afternoon of the same day. The total number of hours allocated for lectures and dissection is 110 and 210 respectively. Typically, students are given a schedule of dissection through region by region using the guidance of a dissection manual (Fig. 1). Faculty are available during the dissection sessions to assist and demonstrate on the clinically important parts or explain the difficult concepts.

The department of Anatomy at CUHAS introduced RPT in teaching Anatomy dissection to first-year medical students (MD1). Because of the shortage of trained anatomists at our institution and an increasing number of students, we chose to institute RPT during Anatomy dissection in order to try to address these challenges. Since this is a form of active learning, it was expected that RPT will improve knowledge and performance in Anatomy, increase the collaboration among class peers, improve student’s communication skills as well as improve the effectiveness of their oral presentations, which is important during their clinical years and during practice. Therefore, this study was conducted in order to evaluate students and faculty opinions on the traditional teaching and reciprocal peer teaching (RPT) methods during anatomy dissection. In addition, comparison of student’s performance on objective examinations prior and after introduction of RPT was done.

## Methods

During the first half of first semester (October to December 2014), all 227 MD1 students were exposed to the traditional teaching method during human Anatomy dissection. During this period, students dissected the upper limbs, thorax, head, neck as well as neuroanatomy. RPT was introduced in the second half of the first semester (January to March 2015) and during this period, students dissected the abdomen, pelvis, perineum and lower limbs. RPT was implemented to augment the teacher-oriented dissection that is usually done at CUHAS. Two objective examinations were administered in each half (i.e. before and after introduction of RPT) to evaluate student’s performance in gross anatomy. Ethical approval was granted from the joint Catholic University of Health and Allied Sciences/Bugando Medical centre ethical review board.

RPT was conducted as follows:

- At the beginning of the anatomy course, students were assigned to groups of 15 with one group per table in the dissection laboratory. Due to the fact that dissection sessions are conducted four days a week, each student had a chance to dissect at least once for one hour every week.
- All students attended a focused lecture on a specific region in the morning before dissecting that region in the afternoon of the same day
- Two students from every table were chosen randomly every day to dissect as well as to teach the peers in their assigned groups under the observation of anatomy faculty. The primary dissectors were taught by anatomy faculty for 45 min (pre-lab sessions) several hours prior to the actual dissection with the help of three-dimensional interactive images and prosected body parts in order to get anatomical overview and methodological details about how to approach the assigned region. Prosections of body parts were done by experienced anatomists. The role of peer learners was to participate actively through observing the dissection process and asking questions or clarifications to the primary dissectors.
- Approximately 75 % of the two hours allocated for dissection per day was a peer-led dissection while the remaining 25 % of the time was used by teachers to clarify the remaining questions from students.
- In order to ensure equal participation, a schedule was prepared by the department’s head to ensure that each student served in alternating roles as a “primary dissector” and a “peer learner”.

Informed consent was obtained from all participants prior to administering debriefing surveys to students regarding merits, demerits and impact of the teaching method used on student’s preparedness prior to dissection, professionalism and communication skills were administered to the students after completion of the traditional method and then after the RPT method of instruction. These tools have been validated before [39]. Since some of the questions asked to students for tradition dissection and RPT protocols were similar, we present these responses together in order to understand which protocol is most preferred by students.

In the survey, students were asked to agree, disagree, or give no opinion. Intensity of agreement was measured using a five-point Likert rating scale [41]. We also assessed student performance on two examinations done before RPT was introduced vs performance on two examinations done after RPT was introduced. All 227 first year medical students in the 2014/15 academic year were tested.

An observation tool for anatomy faculty was used to assess collaboration, professionalism and teaching skills among students. However, because of limitation in time, this tool was not validated prior to its use. The six anatomy faculty moved from table to table to observe individual learners at work and use a scoring guide to track and score observations. This was done as part of the ongoing monitoring system to reflect on the appropriateness of RPT, sharing of information among faculty and assessment of specific students, groups, interactions and the learning environment.

Data was managed using Microsoft Excel spreadsheet and analysis was done using STATA version 12 (College Station, Texas, US). Performance scores were recorded as continuous variables in marks out of 100. We used probability plots and Shapiro-Wilk normality test to assess the normality of performance scores. Performance scores were summarized as mean with standard deviation. For comparison of performance of 2014/15 class before RPT versus after RPT we used paired student’s t-test. The significance of difference in means for RPT and non-RPT performance scores was considered significant if a *p*-value was less than 0.05.

## Results

### Demographic characteristics of students participated in traditional and RPT methods

Of the 227 students in the class, 159 (70 %) and 148 (65.2 %) completed surveys on traditional and RPT methods respectively (Table 1). Female students who completed survey on traditional and RPT methods were 39 % and 43 % respectively. Majority of students (>92 %) who participated both in traditional and RPT surveys had passed advanced certificate of secondary education examination as a qualification to join medical school (Table 1).

**Table 1:** Demographic characteristics of students participated in Traditional and RPT methods

	Traditional (*n* = 159)	RPT (*n* = 148)
Mean age (yrs)	23	23
Sex		
Male	97 (61 %)	84 (57 %)
Female	62 (39 %)	64 (43 %)
Highest educational level		
ACSEE	146 (92 %)	138 (93 %)
Diploma	13 (8 %)	10 (7 %)

### Differences between grades of the same class obtained with and without RPT

A total of 227 students did anatomy examinations before and after RPT was introduced. The mean performance was significantly higher after introduction of RPT compared to the performance before introduction of RPT [63.7 ± 11.4 versus 58.6 ± 10, mean difference 5.1; 95 % CI = 4.0–6.3; *p*-value < 0.0001], see Table 2.

**Table 2:** Mean grades of the same class obtained with and without RPT

Class (*n*)	Mean Score (%)	Std deviation	*p* value
With RPT (227)	63.7	11.4	< 0.0001
Without RPT (227)	58.6	10.8	

In addition to the comparison above, we also determined which group of students, between those who had failed and those passed prior to introduction of RPT, had better improvement in performance after introduction of RPT using two sample student’s t test (see Table 3). There were 185 students who had passed (scored 50 and above) the anatomy examinations before introduction of RPT protocol. The mean score for this group was 62.5 ± 8.2. This group of students improved their performance after introduction of RPT test with the mean score of 66.0 ± 10.4. Thus the mean difference in score before and after introduction of RPT for these students was 3.5 ± 8.1. On the other hand, there were 42 students who had failed (scored below 50) the anatomy examinations before introduction of RPT protocol with a mean score of 42.8 ± 4.8. This group of students improved their performance after introduction of RPT test with the mean score of 53.8 ± 10.1. The mean difference in score before and after introduction of RPT for these students was 11.0 ± 9.5. There was much significant improvement on the group of students who had failed anatomy examination prior to introduction of RPT compared to the group that passed the examination [Mean difference 7.6; *p*-value < 0.0001]. Thus students with low performance prior to RPT benefited more in terms of examination performance compared to those who had higher performance.

**Table 3:** Comparison in improvement of scores between students failed and passed prior to RPT introduction using two sample t test

Students (*n*)	Score prior to RPT	Score after to RPT	Mean difference	Variation of the mean difference	*p*-value
	Mea*n* + SD	Mea*n* + SD			
Passed (185)	62.5 ± 8.2	66.0 ± 10.4	3.5 ± 8.1	7.6	< 0.0001
Failed (42)	42.8 ± 4.8	53.8 ± 10.1	11.0 ± 9.5		

### Student opinions on traditional teaching method

Of the 227 students in the class, 159 (70 %) completed the survey on traditional methods. Table 4 shows that seventy-one percent agreed or strongly agreed that they felt obligated to master the material during the dissection session and the same percentage felt obliged to behave as professionals when working with their groups (respect the rights of team members, cooperation, coordination, support and trust). Student opinions on their interactions and communication during dissection sessions were also assessed. Some of the benefits of the traditional method included enhanced learning of anatomy, more efficient use of time and builds confidence (see Table 4). In addition to these benefits, we also assessed student opinions about major disadvantages of the traditional method. Twenty-two percent of respondents thought that there wasn’t enough time in the lab while 37 % felt that there were too few opportunities to actively dissect. Other drawbacks of the traditional method included inadequate teaching from peers and overcrowding of students in a dissection table (21 and 17 % respectively). None of the student had a prior experience with traditional dissection method.

**Table 4:** Student opinions about Traditional teaching method

Opinion	Response (%)
Effect of the current dissection practice on my gross	
Anatomy education	
Strongly negative	1
Mildly negative	11
Neutral	5
Mildly positve	33
Strongly positive	50
The greatest benefit of the current dissection practice	
Enhanced learning anatomy	71
More efficient use of time	17
Experience of teaching peers	6
No benefits of traditional method	2
Builds confidence	4
The greatest drawback of the current dissection practice	
Not enough time in lab	22
Few opportunities to dissect	37
Didn’t receive adequate teaching from Peers	21
Prior experience with traditional dissection method	
Yes	0.8
No	99.2

### Student opinions about reciprocal peer teaching method

Of the 227 students in the class, 148 (65.2 %) completed the survey on RPT protocol. When students were “primary dissectors”, the majority (75 %) strongly agreed or agreed that they felt obligated to master the material during the dissection session, because they were responsible for teaching their peers (see Table 5). Regarding professionalism, (79 %) of the students agreed or strongly agreed that they felt obliged to behave as professionals when working as a primary dissectors or peer learners. When asked if alternating schedule during RPT prevented them from dissecting all the parts they wanted, 52 % either disagreed or strongly disagreed with that assertion. An overwhelming majority (91 %) reported that the 45 min pre-lab demonstration improved their anatomy knowledge as primary dissectors. Eighty-eight percent of students felt that RPT had a positive effect on their learning and therefore agreed or strongly agree that RPT should be continued. Some of the benefits of RPT that were mentioned by respondents in order of importance included enhanced learning of anatomy, experience of teaching peers, more efficient use of time and building confidence among students. Only 1 % of respondents stated that RPT had no benefits. On the disadvantages of RPT protocol, 25 % of respondents thought that there wasn’t enough time in the lab while 48 % felt that there were too few opportunities to actively dissect. Other drawbacks of the RPT protocol included inadequate teaching from peers and overcrowding of students in a dissection table (7 and 12 % respectively).

**Table 5:** Student opinions about Reciprocal Peer Teaching

Opinion	Response (%)
Alternating schedule prevented me from dissecting all the parts I want to	
Strong disagree	9
Disagree	43
Neither agree nor disagree	4
Agree	32
Strong agree	12
Pre-lab demonstration improved my knowledge as a primary dissector	
Strong disagree	3
Disagree	3
Neither agree nor disagree	3
Agree	45
Strong agree	46
Effect of Reciprocal Peer Teaching on my gross anatomy education	
Strongly positive	51
Mildly positive	37
Neutral	11
Mildly negative	1
Strongly negative	0
The greatest benefit of RPT	
Enhanced learning of anatomy	59
More efficient use of time	10
Experiencing of teaching peers	26
There are no benefits of RPT	1
Other, please specify (builds confidence)	4
The greatest drawback of RPT	
Not enough time in the lab	25
Few opportunities to actively dissect	48
Didn’t receive adequate teaching from peers	7
There are no drawbacks of RPT	8
Too many people in one group	12
Other comments related specifically to RPT in the anatomy lab	
1. Every student should have opportunity to play both roles	
2. RPT should be maintained at CUHAS	
3. Dissection groups should be smaller for easy teaching and understanding	
Prior experience with Reciprocal Peer Teaching method	
Yes	0
No	100

The survey also included a space for personalized comments regarding RPT. Some students commented “RPT should be maintained at CUHAS”, while others commented, “every student should have an opportunity to play both roles.” Other students recommended that dissection groups should be smaller for easy teaching & understanding. None of the student had a prior experience with Reciprocal peer teaching method.

### Student’s opinions on traditional dissection practice vs. Reciprocal peer teaching

**Fig. 2:**
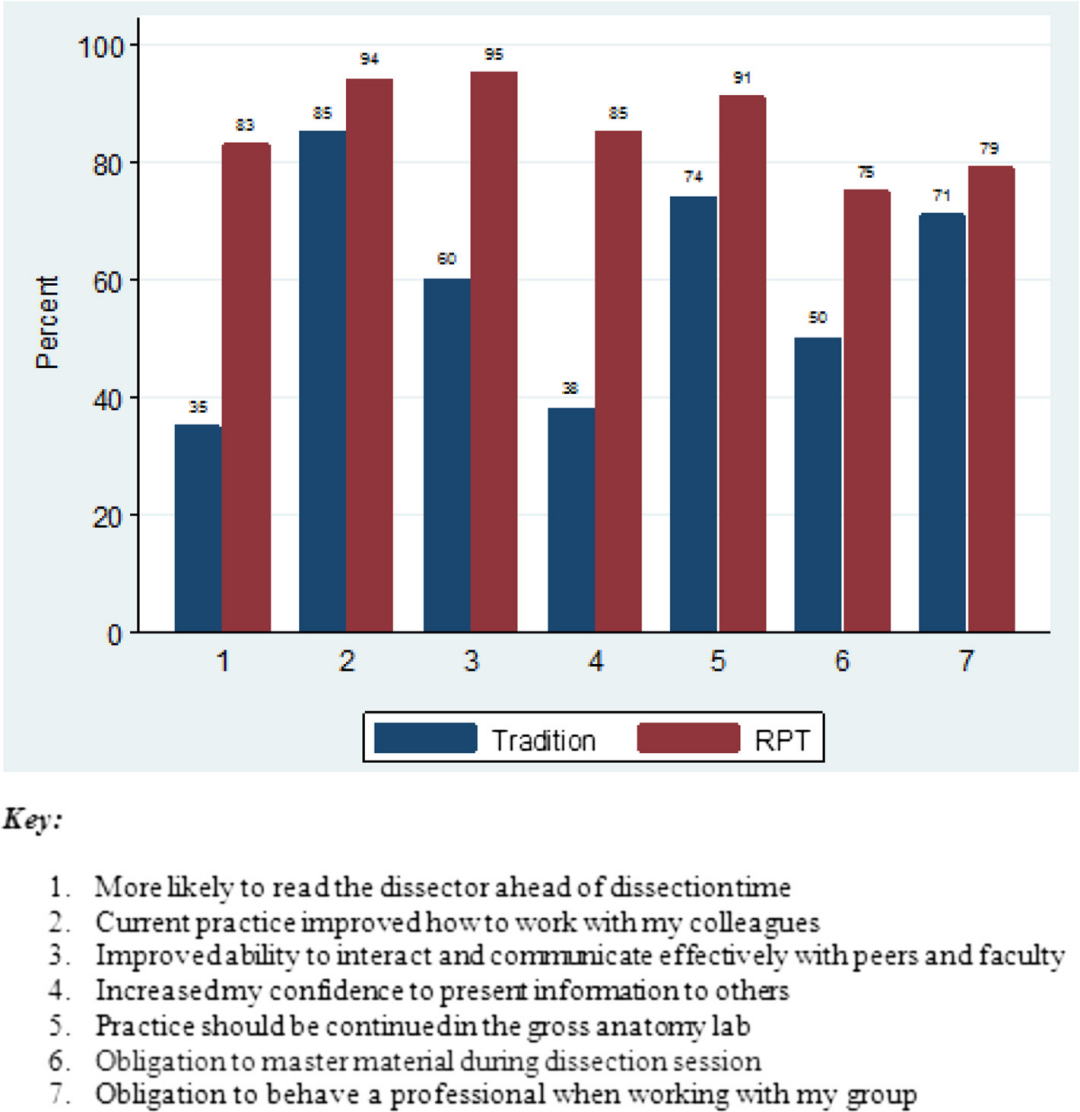
Student’s opinions. A Histogram showing student’s opinions on the Traditional dissection practice versus Reciprocal Peer Teaching

The opinions pertained to student’s preparedness prior to dissection, professionalism, working as teams and communication skills. Most (83 %) of students either agreed or strongly agreed that they were more likely to read the dissection manual before the RPT dissection session compared to 35 % for the traditional method (Fig. 2). The majority (95 %) of students either agreed or strongly agreed that their ability to interact and verbally communicate effectively with peers and faculty was improved after RPT sessions compared to 60 % for tradition method. Over 85 % of respondents reported that RPT improved their confidence and ability to present information to peers and faculty compared to 38 % for the tradition method.

Similar trend of results were observed on other aspects i.e. ability to work with colleagues and if the protocol should be continued.

### Faculty opinions regarding student’s conduct during RPT sessions

**Fig. 3:**
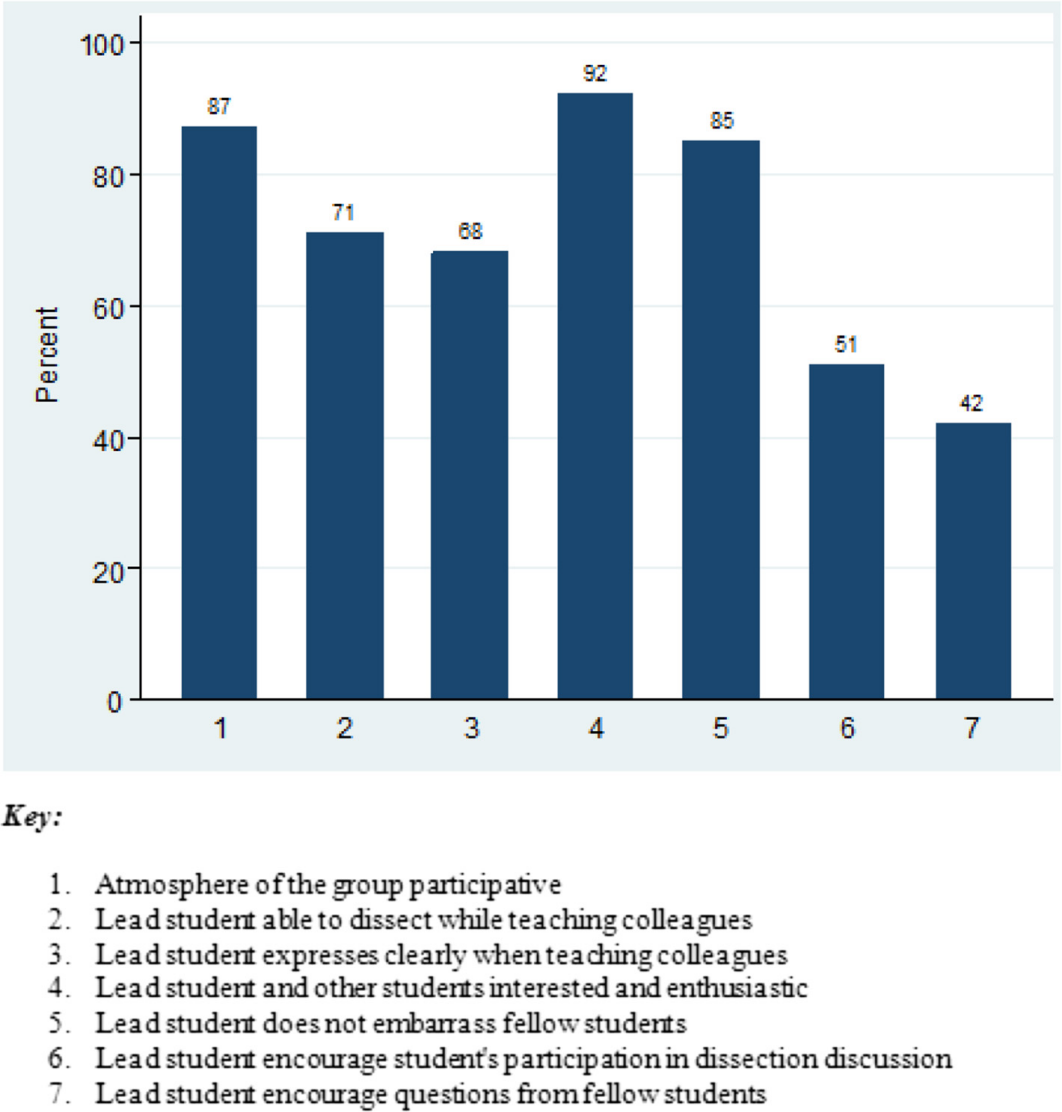
Faculty opinions. A Histogram showing teacher’s opinions regarding student’s conduct during Reciprocal Peer Teaching sessions

The six anatomy faculty assessed collaboration, professionalism and teaching skills among students during RPT sessions. More than 80 % of faculty reported that the atmosphere of the dissection groups were very participative during RPT sessions while 72 % of faculty thought that peer dissectors were able to dissect while teaching peers (Fig. 3). Most of faculty (93 %) reported that peer instructors and students were interested and enthusiastic during the RPT dissection sessions. The majority of faculty (84 %) reported that peer instructors did not act or behave in a way that embarrassed fellow students. Faculty also reported that peer instructors encouraged student’s participation in discussions during dissection and encouraged questions from fellow students respectively (Fig. 3).

## Discussion

In an effort to address the shortage of doctors and other health care workers in the Tanzania, the number of students enrolling in each first year class at CUHAS has increased significantly from 10 students in 2003 to 220 students in 2014. This increase, without a concomitant rise in faculty numbers, has led to a shortage of anatomy faculty capable of adequately moderating dissection sessions. The traditional approach to anatomy course conducted at CUHAS prior to introduction of RPT is equally similar to some of the anatomy programs in other medical schools [2, 42, 43] but also different from others [13, 44]. The high student/faculty ration during dissection observed in this study is pretty similar to some of the other medical schools in Africa [2, 3, 42]. Majority of students participated in both surveys were males, which reflects the composition of each gender in the class and at most of the medical schools in Tanzania. Students participated in both surveys who had a diploma in clinical medicine as a qualification to join medical school were less than 10 %. During training for a diploma in clinical medicine, students are taught basic anatomy but which doesn’t include cadaver dissection. Therefore, the fact that all the students participated in both surveys had no prior exposure to cadaver dissection, avoided the bias of previous exposure to dissection.

The present study was inspired by the success of the peer teaching programmes in practical gross anatomy at several universities across the globe where RPT has been shown to provide students with ample time for independent learning and address the challenge of shortage of anatomy faculty during dissection sessions [35, 36, 38–40].

Our findings indicate that there were significant improvements in the examination mean scores of students of the same class after the introduction of the RPT exercise during cadaver dissection. The improvement in performance of anatomy examinations is likely a result of a combination of several factors. First, the fact those students were initially exposed to the traditional method and later to the RPT protocol, might have brought in time and experience as factors that influenced student performance. Often students struggle during the initial duration in the anatomy lab, however, they become more comfortable over time. Our experience from archived anatomy examination results show that students perform poorly on neuroanatomy, head and neck regions compared to other body regions. The fact that these regions were dissected and examined before introduction of RPT, could have contributed to student’s lower performance. It is also possible that, student’s performance was improved by the positive impact of RPT on the knowledge of students, which in turn was reflected by better scores. At all academic levels, the process of teaching others results in a 90 % retention rate of material, as compared to the 5 % for lecture, 10 % for reading, and 50 % for discussion [45]. It is therefore highly likely that knowledge retention by students was improved through RPT participation since every student had a chance to teach peers. Improvement in grades for reading and gross anatomy after introduction of RPT has also been reported from other studies [32, 35, 36].

Of particular interest, was the fact that there was significant improvement in scores of students who failed the pre-RPT examinations compared to those who passed. This could be interpreted that RPT had a bigger impact on weak students. Studies on Team Based Learning protocol, which is a teaching protocol that promotes student engagement in learning anatomy similar to RPT, have shown that it mostly benefits academically the at-risk students who are forced to study more consistently, are provided regular feedback on their preparedness and given the opportunity to develop higher reasoning skills [46–48]. Weaker students might have benefited more by regarding their peers as comfortable learning resources and therefore were more likely to ask peers for help during RPT sessions.

Analysis of the student’s opinions gathered during this study indicates that majority of our student respondents had positive experiences with both the Traditional dissection and RPT protocols. However, the level of positivity for similar questions asked for the two protocols was different with RPT being the most preferred protocol. More than 50 % of student participants stated that both traditional and RPT protocols improved their ability to communicate, interact and work with their colleagues. Similarly, most students reported that their confidence to present materials to peers was improved. Ability to communicate, interact, work with their colleagues and confidence to present materials to peers are among the most important competencies for medical students and medical doctors [49]. Communication is one of the core clinical skill requiring continuous training, practice and feedback during and after medical school training [50]. Training and practices that improves student’s communication skills in medical schools is likely to shape their perception of communication issues and improve their interactions with patients throughout their career [50, 51]. Good communication and collaboration between health care practitioners are essential for good medical care, particularly in the context of inpatient treatment. Literature shows that a poor communication skill among physicians contributes to dissatisfaction and lack of compliance observed among patients [52, 53]. The World Health Organisation (WHO) recommends that teaching teamwork skills should be a central aim of medical education [54]. It is therefore important that the process of imparting effective communication skills among doctors should start early during their training.

The majority of students recommended that both practices should be continued for future classes. This could be due to the fact that traditionally, most of the teaching strategies used in Tanzania from elementary school to University are passive teaching styles and teacher-centered. Both tradition dissection and RPT practice are active learning processes which keep the student absorbed and interested till the end [55]. It is highly likely that introduction to dissection practice (either traditional or RPT) might have been the first exposure to active teaching student-centered style. Similar explanations could explain the findings that majority of student respondents felt that the effect of both traditional and RPT protocols on their gross anatomy education was either mildly or strongly positive. Dissection classes has generally been highly valued, utilized more effectively and improves teamwork among students [56]. Findings from other studies that evaluated the impact of student-centered teaching methods in gross anatomy have shown that, these methods significantly improve student performance in examinations [57, 58]. However, in certain contexts, including the context of compressed learning time, the student-centered approach may have negative impacts on learning (as measured by student performance on exams) that may offset the accepted benefits of student-centered approaches [28].

Subjective opinions from students have proven very informative with respect to the duration of dissection and optimum number of students per each dissection table/cadaver. Majority of students opined that the time and opportunity to actively dissect were not enough and that dissection groups should be smaller for easy teaching & understanding. We didn’t probe further on student’s recommendation on the duration for anatomy dissection and the optimal number of students per dissection table/cadaver. Elsewhere, it has been shown that a team of three to four students per cadaver is adequate to carry out dissection tasks [39]. A combination of utilizing RPT, increased dissection space and more cadavers can help address the concern of overcrowding in the dissection laboratory. Some of the medical schools across the globe conduct human cadaver dissection in two academic semesters whereby dissection of the limbs, thorax, abdomen and pelvis regions is conducted in first year while dissection of the head and neck region is conducted in second year of MD program [42, 43]. This arrangement helps to provide more time for thorough dissection and better understanding of the subject. The current curriculum review of the MD program at CUHAS could address this challenge.

The positive reception of RPT by students was also echoed by faculty observations. Faculty reported that students were engaged, interested and enthusiastic during the RPT dissection sessions. In addition, the level of professionalism among students was very high. Findings from both faculty and student’s opinions showed that RPT is a useful approach in promoting student engagement in learning anatomy. The department of anatomy at CUHAS, depending on the availability of necessary resources, will consider to introduce the other methods that promotes students engagement including team-based gamification [59], collaborative practical examinations [60], Digital team reporting [61] and formative assessment [62]. Despite the fact that results from this study provides valuable information about tradition and RPT protocols during dissection sessions, this study provides several limitations. First, we did not achieve participation from all of our students both for tradition and RPT surveys. This could have been caused by the nature of the survey (which was voluntary) and also the fact that the surveys were done during the short academic breaks in December and March when some of the students were away from campus. Evidence from literature however shows that, high response rates do not necessarily predict accurate attitudes [63]. Therefore it’s our belief that the respondent group captured in our study was representative enough for the target population. Second, because of the anonymous nature of the questionnaire, we could not correlate the nature of responses and examination performances. Thirdly, the tool used by faculty to assess student’s participation during RPT practice was not validated prior to their use. This could have lead to some important information being missed. In addition, the fact that no training was provided to faculty participating in student’s observation in terms of normalizing observations and the same faculty being co-authors in this paper, could provide a potential for bias in their responses.

Lastly, we’re unable to compare final examination scores of students in 2014/15 trained both by RPT and traditional methods with the previous classes trained by tradition method because of some disparities including, difference in size of class between 2014/15 class and the previous classes. Some of the previous examination included both objective (multiple choice questions) and subjective (essays questions) assessments while only objective assessment was used for 2014/15 class. In addition, the previous classes were trained entirely on traditional method and therefore could not be compared fairly with 2014/15 class that was trained with both methods.

## Conclusions

Introduction of RPT in our anatomy dissection laboratory was generally beneficial to both students and faculty. Both objective (student’s scores) and subjective data indicate that RPT improved student’s performance and had a positive learning experience impact. In addition, RPT introduced to our students an experience of peer teaching, this improved their confidence and communication skills that they can apply throughout their careers. The active involvement of students in teaching will help prepare students to be continual and independent self-learners throughout their professional careers. The practice of pre-lab sessions helped to create “peer instructors” who helped to teach their peers and therefore helping to address the shortage of anatomy faculty during dissection sessions.

Our future plans are to continue RPT practice and continually evaluate the RPT protocol. We will also assess faculty satisfaction with this teaching method and make modifications based on student and faculty feedback and trends in available resources at the university.

### Ethics approval and consent to participate

Informed consent was obtained from all participants prior to data collection. The ethical approval was obtained from the joint Catholic University of Health and Allied Sciences and Bugando Medical Centre (CUHAS/BMC) ethical review board.

### Availability of data and materials

Raw data will not be shared due to restrictions stipulated by the joint Catholic University of Health and Allied Sciences and Bugando Medical Centre (CUHAS/BMC) ethical review board that approved this study.
